# Is percutaneous proximal gracilis tenotomy as effective and safe as the open procedure?

**DOI:** 10.1007/s11832-015-0699-z

**Published:** 2015-10-26

**Authors:** Bilal Hachache, Tony Eid, Elias Ghosn, Amer Sebaaly, Khalil Kharrat, Ismat Ghanem

**Affiliations:** Department of Orthopaedic Surgery, Hôtel-Dieu de France Hospital, Saint-Joseph University, Alfred Naccache Street, Ashrafieh, Beirut, Lebanon

**Keywords:** Proximal gracilis lengthening, Proximal gracilis tenotomy, Percutaneous tenotomy, Subcutaneous tenotomy, Tendon lengthening, Cerebral palsy hip

## Abstract

**Purpose:**

There is currently an increasing trend for percutaneous surgical interventions mainly in children with cerebral palsy (CP). The purpose of this study was to evaluate the effectiveness and safety of percutaneous proximal gracilis tenotomy (PPGT) in children with CP scheduled for hip adductor tenotomy.

**Methods:**

This is a prospective study of 59 hips in 31 consecutive patients with CP scheduled for hip adductor tenotomy in the setting of multilevel tenotomies or hip osteotomy (femoral or Dega). A pediatric orthopedic surgeon conducted a percutaneous adductor longus and gracilis tenotomy through the same stab wound. Another surgeon extended the wound to explore what had been cut during the PPGT, and completed the tenotomy if necessary (open proximal gracilis tenotomy; OPGT). Hip abduction with the hip and knee extended (HA) was assessed by a third surgeon (1) immediately before PPGT, i.e., directly after percutaneous adductor longus tenotomy (prePPGT), (2) after PPGT (postPPGT), and (3) following OPGT (postOPGT), using a goniometer, in a standardized reproducible manner. All three surgeons were blinded to each other’s findings. Primary end-points included the percentage of muscle portion sectioned percutaneously and the improvement of HA angle. Comparison between HA before and after PPGT was performed using a paired *t* test with 95 % confidence interval (CI), and comparison between HA after PPGT and OPGT was performed using a Student’s *t*-test with 95 % CI. The bleeding was assessed and other iatrogenic lesions were identified. The relationship between HA after PPGT and the percentage of muscle portion sectioned percutaneously was evaluated by calculating the Pearson correlation coefficient (*p* < 0.01).

**Results:**

Mean HA measured 33.71 degrees prePPGT and increased to 45.90 degrees postPPGT (*p* < 0.0001). The postOPGT HA averaged 48.71 degrees with no statistically significant gain compared with postPPGT (*p* = 0.21). The muscular portion of gracilis origin was cut to an average of 91.95 %; completely in only 14 hips, between 90 and 100 % in 35 hips, between 70 and 90 % in 9 hips, and between 60 and 70 % in 1 hip. The gain in HA did not correlate with the extent of the muscular portion sectioned percutaneously (*R* = −0.043). Minimal accidental section of adductor brevis postPPGT was encountered in 39 hips. Considerable bleeding postPPGT with hematoma formation requiring hemostasis during the open control procedure occurred in 30 hips. Partial iatrogenic injury of the anterior branch of the obturator nerve was encountered in one patient bilaterally with severe adductor contracture, due to an anatomic too medial variant.

**Conclusions:**

This is the only prospective study concerning the outcome of PPGT. Although PPGT is fast, simple and effective, it is not as safe as the open procedure even when performed correctly by an experienced surgeon, mainly because of the increased risk of bleeding. The findings of the current study do not support its use as a ‘standard-of-care’ technique in children with hip adductor contracture.

**Level of evidence:**

Level II therapeutic study—prospective comparative study.

## Introduction

Spasticity and contracture of hip adductors in cerebral palsy (CP) may interfere with walking or lead to gradual hip dislocation. Although many conservative treatment modalities exist, including physical therapy, positioning and botulinum toxin injection, tenotomy is often required [1]. Walking ability, as defined with use of the Gross Motor Function Classification System (GMFCS) level, is a strong predictor of success or failure after hip adductor surgery in children with CP. The paradox of hip adductor surgery for children with CP is that the children who are most severely affected and need the surgery the most have the poorest results [2].

Adductor longus and gracilis are most frequently addressed and surgery is usually performed through a 2–3 cm obturator incision [3–5]. There is currently an increased trend for percutaneous multilevel tendon or even bone procedures in CP based on their reported advantages [6, 7]. El Hage et al. showed that when performed correctly in patients with CP, percutaneous adductor longus tenotomy is fast, simple, reliable, effective and safe [7]. Percutaneous proximal gracilis tenotomy (PPGT) is a rarely used procedure, and to the best of our knowledge has never been reported or described in the literature.

The purpose of this study was to evaluate the effectiveness and safety of PPGT and to compare it with the open procedure (OPGT).

## Methods

This is an IRB-approved prospective study of patients with CP who were scheduled for multilevel tenotomies (psoas over the brim and/or rectus femoris, adductor longus, gracilis, medial hamstrings, gastrocnemius or Achilles) or hip osteotomy (adductor and psoas tenotomy, femoral osteotomy with or without Dega osteotomy) over a period of 2 years. Each hip received both the percutaneous followed by the open procedure and is thereby considered as case and control at the same time.

**Fig. 1:**
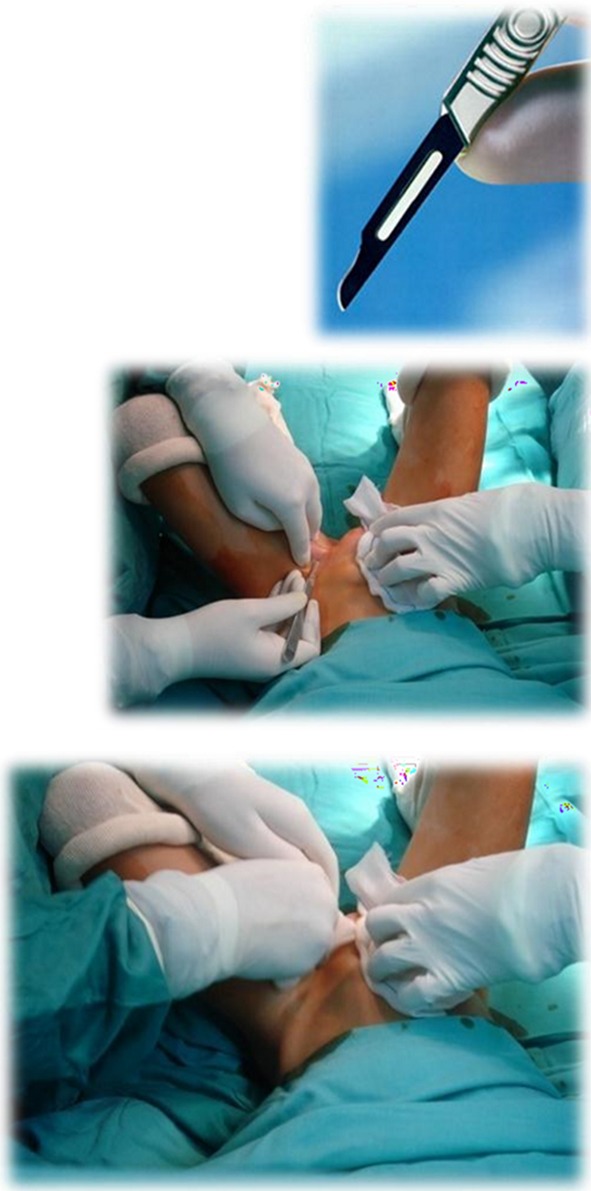
Standardized technique for PPGT. **a** Surgical blade used, **b** patient position and tenotomy, **c** manual back and forth movements on the surgical site, applying pressure with a gauze

Three independent surgeons participated in the study. The first surgeon, right-handed, carries out the PPGT in a standardized way. He stands to the patient’s left side even in bilateral cases, and with the patient’s hips abducted he performs an obturator stab wound over the adductors prominence through which he undertakes first the adductor longus tenotomy followed by the gracilis. Any remaining fibers are then divided in a closed manner by back and forth movements while applying pressure with a gauze (Fig. 1). All bony procedures (femoral or pelvic osteotomies) and soft-tissue procedures (specifically, psoas, rectus femoris and hamstrings tenotomies) were performed after hip measurements.

A second surgeon then approaches the surgical field and extends the wound blinded to the first surgeon and vice versa, to explore what has been actually cut during PPGT. He calculates the percentage of muscular fibers cut as the ratio between the extent of the proximal percutaneously divided gracilis portion and the entire extent of the proximal gracilis insertion using a flexible ruler. He also looks for iatrogenic injury to surrounding vessels or the obturator nerve, and to neighboring muscles (adductor brevis, adductor magnus), and undertakes necessary measures to stop any bleeding. Finally, this surgeon completes the gracilis tenotomy when necessary using electrocautery, closes the wound in two layers and proceeds with other tenotomies or osteotomies elsewhere on the patient’s lower limbs depending on the patient’s history and preoperative planning.

**Fig. 2:**
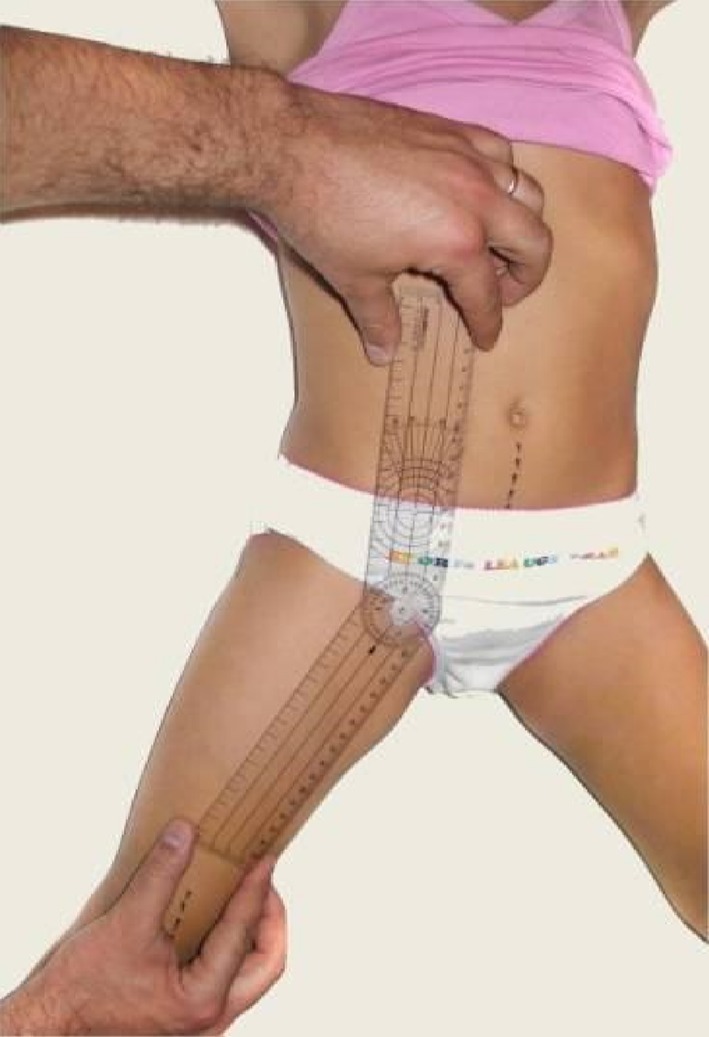
Standardized reproducible manner for hip abduction measurements using a calibrated goniometer

The third surgeon blinded to the two previous surgeons, is responsible for making HA measurements, separately for each side, using a 1-degree calibrated goniometer for both hips in hip and knee extension, after controlling the pelvic motion carefully. The first one is performed after percutaneous adductor longus tenotomy and immediately before percutaneous gracilis tenotomy, and is considered as the preoperative HA angle for gracilis tenotomy (prePPGT). The second measurement is undertaken directly after PPGT (postPPGT), and the third immediately following OPGT (postOPGT) (Fig. 2).

The duration of both the PPGT and OPGT was also recorded.

Comparison between HA prePPGT and postPPGT was performed using a paired *t*-test with a 95 % confidence interval (CI), and comparison between HA postPPGT and postOPGT was done using a Student’s *t*-test with a 95 % CI. The relationship between HA after PPGT and the percentage of muscle portion divided percutaneously were evaluated by calculating the Pearson correlation coefficient (*p* < 0.01).

## Results

Thirty-one consecutive patients (16 males and 15 females) with 59 hips were included in the study. The mean age at surgery was 8.5 years ± 3.4 SD, and the GMFCS level was I in 6 cases, II in 14 cases, III in 24 cases, IV in 10 cases, and V in 5 cases. Adductor tenotomy was performed in the setting of single-event multilevel tenotomies in 50 hips and as part of the treatment of a hip subluxation or dislocation (femoral varus derotational osteotomy with or without San Diego-Dega osteotomy) in the 9 remaining hips.

**Fig. 3:**
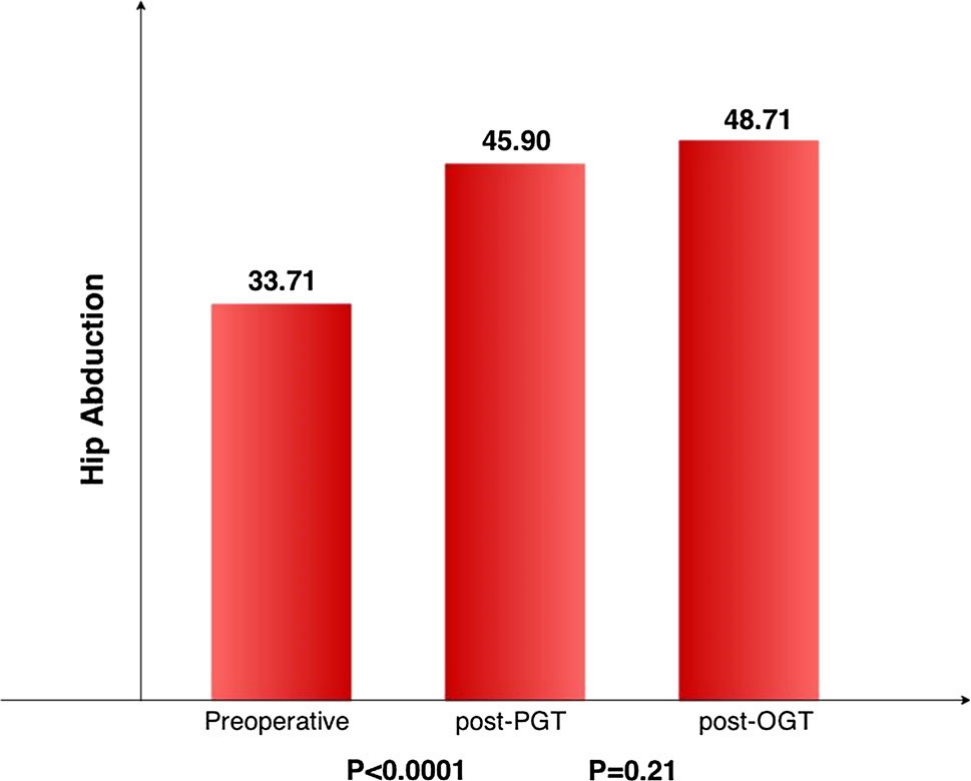
Hip abduction values after percutaneous (PPGT) and open (OPGT) procedures

The mean prePPGT HA was 33.71° ± 9.58 SD and increased to 45.90° ± 11.60 SD postPPGT (*p* < 0.0001) and 48.71° ± 12.75 SD postOPGT (*p* = 0.21) (Fig. 3). The muscular portion of gracilis origin was cut to an average of 91.95 % (completely in only 14 cases, between 90 and 100 % in 35 cases, between 70 and 90 % in 9 cases, and between 60 and 70 % in 1 case). The gain in HA did not correlate with the extent of the muscular portion sectioned percutaneously (*R* = −0.043).

**Fig. 4:**
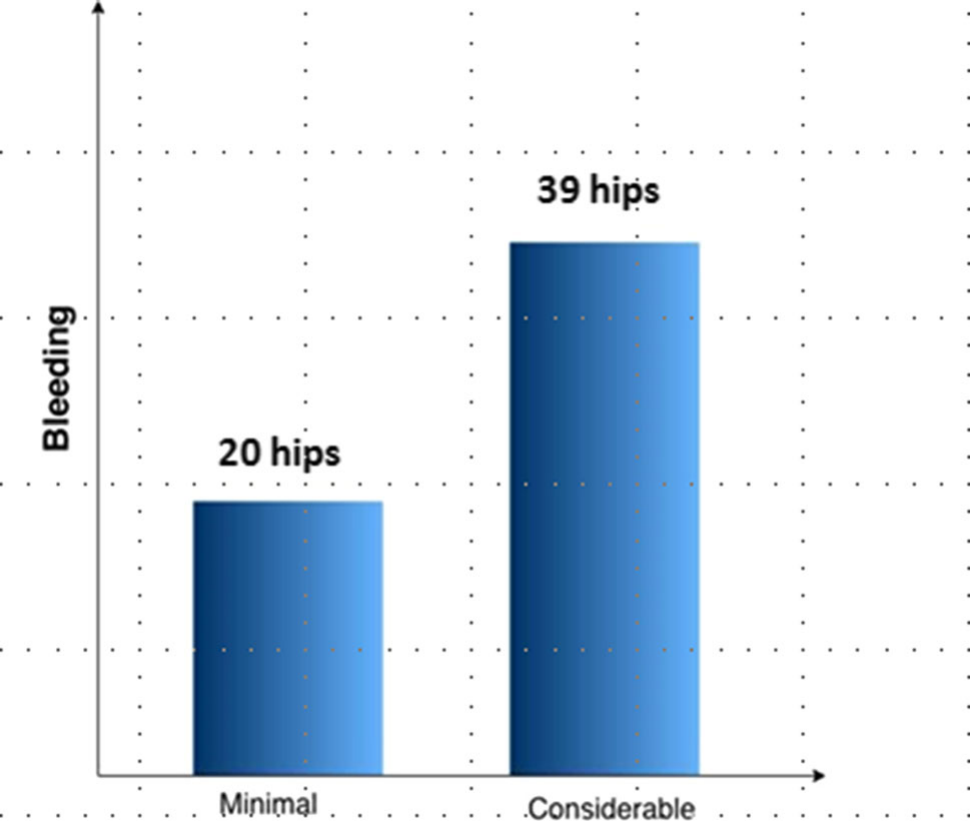
Bleeding evaluation after PPGT. ‘Minimal’ for mild and moderate bleeding not requiring hemostasis other than pressure from a gauze at the surgical site; ‘Considerable’ for bleeding that does not stop by compression alone and requires electrocautery for hemostasis

Very minimal accidental injury of adductor brevis after PPGT was encountered in 39 cases. Considerable bleeding, i.e., bleeding that does not stop by compression alone, or hematoma formation occurred in 39 hips (66 %), 30 (51 %) of which required scrupulous hemostasis during the open control procedure (Fig. 4). Partial iatrogenic injury of the anterior branch of the obturator nerve was encountered in one patient bilaterally with very severe adductor contracture, due to an anatomic too medial variant of the nerve trajectory. The same patient had also a minimal partial injury of the adductor brevis.

The duration of PPGT ranged from 12−17 s (average 15 s), whereas OPGT lasted 5–8 min (average 7 min) including wound closure and hemostasis when needed.

## Discussion

To the best of our knowledge, no study has evaluated the PPGT or compared its effectiveness and safety with those of OPGT to date. Although many series have been published dealing with adductor release in the CP setting, the vast majority comprise heterogeneous populations who have undergone surgery by both percutaneous and open procedures without distinction, with numerous associated procedures (obturator neurotomy, psoas tenotomy, osteotomies) making proper analysis and conclusions difficult [4, 8–10]. Moreover, the PPGT has rarely been used and has never been described in textbooks and surgical atlases, and its anatomic basis has never been clearly illustrated.

The effectiveness and safety of other percutaneous tendinous procedures have been evaluated. Thompson et al. considered that minimally invasive single-event multilevel surgery can be achieved safely and effectively with significant advantages over conventional techniques in children with diplegic CP [6]. Adductor longus tendon and Achilles tendon tenotomies and lengthenings have recently been studied. El Hage et al. showed that percutaneous adductor longus tenotomy is fast, simple, and as reliable and effective as the open procedure without any major risks [7]. Mulier et al. described a case of a false aneurysm that developed after a repeat percutaneous Achilles lengthening [11]. Salamon et al. carried out cadaveric triple hemisection percutaneous Achilles tendon lengthenings [12]; they found that percutaneous Achilles tendon lengthening is a relatively accurate procedure with hemisections averaging 50 % for the middle cut and 60 % at the most proximal cut, and 55 % at the distal cut. Some tendinous and neurovascular structures are, on average, <1 cm from the nearest margin of a given hemisection and are, therefore, at risk including the flexor hallucis longus, the tibial nerve, and the sural nerve. More recently, Hoefnagels et al. in another cadaveric study showed that the percutaneous triple hemisection technique results in more technical failures than acceptable and suggests carrying out the technique as an open procedure [13].

Vascular complications after percutaneous Achilles tenotomy in clubfoot management using the Ponseti method have also been reported in three cases with serious bleeding from injury to the peroneal artery and another case of injury to the lesser saphenous vein [14]. The authors detail the technique of carrying out a percutaneous heel cord tenotomy and offer guidelines that may help others avoid this same complication. Another recent report described a pseudoaneurysm after percutaneous Achilles tendon tenotomy in a clubfoot treated with the Ponseti method [15].

With no clear technique for PPGT and no direct visual control, one may worry about accidental injury to nearby structures such as the femoral vein or the anterior branch of the obturator nerve, especially because of the large and deep insertion of the gracilis on the body and inferior pubic ramus. Moreover, the muscular localization of the tenotomy due to the very small tendinous insertion of the gracilis, may be the cause of bleeding and hematoma. According to the vascular anatomy of the obturator region, the origin of bleeding could be the superficial external pudendal vessels.

Evaluation of the long-term effect of PPGT on abduction gain in comparison with OPGT was beyond the scope of our objectives. Nevertheless, we believe that there should be no difference between the two procedures in terms of long-term gain in abduction and other endpoints such as prevention of hip subluxation and pain.

Some recent reports show nonreproducible and sometimes dangerous effects of percutaneous tendon procedures [11–15]. Our results seem to indicate that percutaneous proximal gracilis tenotomy achieves similar results on short-term abduction gain as its open counterpart, but even when performed correctly by an experienced pediatric orthopedic surgeon is not as safe as the open release mainly because of the increased risk of bleeding, and is even more contraindicated in less experienced hands. In our opinion, the data in this study offer sufficient evidence to contraindicate PPGT.

## References

[1] Fettweis E (1979). Spasm of the adductor muscles, pre-dislocation and dislocations of the hip joints in children and adolescents with cerebral palsy. Clinical observations on aetiology, pathogenesis, therapy and rehabilitation. Part I: the effect of open myotenotomy of the gracilis muscle and of the long and short adductor muscles in connection with total extrapelvine resection of the obturator nerve, on the hip joints and static function. Z Orthop Ihre Grenzgeb.

[2] Shore BJ, Yu X, Desai S (2012). Adductor surgery to prevent hip displacement in children with cerebral palsy: the predictive role of the gross motor function classification system. J Bone Joint Surg Am.

[3] Bishay SN (2008). Short-term results of musculotendinous release for paralytic hip subluxation in children with spastic cerebral palsy. Ann R Coll Surg Engl.

[4] Cottalorda J, Gautheron V, Metton G (1998). Predicting the outcome of adductor tenotomy. Int Orthop.

[5] Rolauffs B, Stuby F, Barth S (2007). Prophylaxis and therapy for hip dislocations in patients with infantile cerebral palsy (ICP): motor functional, radiological and clinical results after subcutaneous adductor tenotomy. Z Orthop Unfall.

[6] Thompson N, Stebbins J, Seniorou M (2010). The use of minimally invasive techniques in multi-level surgery for children with cerebral palsy. J Bone Joint Surg Br.

[7] El Hage S, Rachkidi R, Noun Z (2010). Is percutaneous adductor tenotomy as effective and safe as the open procedure?. J Pediatr Orthop.

[8] Bagg MR, Farber J, Miller F (1993). Long-term follow-up of hip subluxation in cerebral palsy patients. J Pediatr Orthop.

[9] Cornell MS, Hatrick NC, Boyd R (1997). The hip in children with cerebral palsy. predicting the outcome of soft tissue surgery. Clin Orthop Relat Res.

[10] Rutz E, Vavken P, Camathias C (2015). Long-term results and outcome predictors in one-stage hip reconstruction in children with cerebral palsy. J Bone Joint Surg Am.

[11] Mulier T, Molenaers G, Fabry G (1995). A false aneurysm complicating a subcutaneous Achilles tendon lengthening. J Pediatr Orthop B.

[12] Salamon ML, Pinney SJ, Van Bergeyk A (2006). Surgical anatomy and accuracy of percutaneous achilles tendon lengthening. Foot Ankle Int.

[13] Hoefnagels EM, Waites MD, Belkoff SM (2007). Percutaneous Achilles tendon lengthening: a cadaver-based study of failure of the triple hemisection technique. Acta Orthop.

[14] Dobbs MB, Gordon JE, Walton T (2004). Bleeding complications following percutaneous tendoachilles tenotomy in the treatment of clubfoot deformity. J Pediatr Orthop.

[15] Burghardt RD, Herzenberg JE, Ranade A (2008). Pseudoaneurysm after Ponseti percutaneous Achilles tenotomy: a case report. J Pediatr Orthop.

